# A Comparative Study on Color Stability of Anthocyanin Hybrid Pigments Derived from 1D and 2D Clay Minerals

**DOI:** 10.3390/ma12203287

**Published:** 2019-10-10

**Authors:** Shue Li, Bin Mu, Xiaowen Wang, Yuru Kang, Aiqin Wang

**Affiliations:** 1 Key Laboratory of Clay Mineral Applied Research of Gansu Province, Center of Eco-Materials and Green Chemistry, Lanzhou Institute of Chemical Physics, Chinese Academy of Sciences, Lanzhou 730000, China; seli17@licp.cas.cn (S.L.); wxw1201@163.com (X.W.); yurukang@licp.cas.cn (Y.K.); 2 Center of Materials Science and Optoelectronics Engineering, University of Chinese Academy of Sciences, Beijing 100049, China; 3 Center of Xuyi Palygorskite Applied Technology, Lanzhou Institute of Chemical Physics, Chinese Academy of Sciences, Xuyi 211700, China

**Keywords:** clay minerals, anthocyanin, hybrid pigments, color stability, pH test paper

## Abstract

Anthocyanin extracted from the fresh blue berry fruits was loaded onto different clay minerals including one-dimensional tubular halloysite and fibrous sepiolite, and two-dimensional lamellar kaolinite and montmorillonite to fabricate reversible allochroic hybrid pigments. The effect of the possible interaction mechanism between anthocyanin and clay minerals on the color stability of hybrid pigments was investigated. Due to the difference in the structures and properties of clay minerals, natural anthocyanin was inclined to be absorbed on the surface and intercalated into the interlayer of 2:1 type layered montmorillonite, while it was mainly anchored on the surface of 1:1 type kaolinite and halloysite. By contrast, it was simultaneously loaded on the surface and confined into the nanochannels and/or grooves of 2:1 type chain-layered sepiolite. Interestingly, the resulting hybrid pigments presented good thermal stability and resistance to chemical reagents, as well as reversible gas-sensitive allochroic behavior in HCl or NH_3_ gases, especially anthocyanin/sepiolite hybrid pigments due to the shielding effect of the well-defined nanochannels and grooves of sepiolite. Based on this color-change behavior, a simple pH test paper was also prepared with obvious color change at different pH values by coating the filter paper with anthocyanin/sepiolite hybrid pigments.

## 1. Introduction

Anthocyanin (ACN) mainly derives from blueberries, grape skins, purple sweet potatoes, acerola juice, and red cabbage [1,2,3,4,5,6,7,8]. In addition, ACN also accumulates in the foliage of various plants, such as liverwort and *Prunuscerasifera* [9]. This water-soluble pigment can impart a variety of vivid colors to above fruits, vegetables, and plants as natural colorants. In other words, the stimulation of the external environments (e.g., pH and temperature) leads to the diversity of ACN color ranging from blue to red, which mainly attributes to the number of hydroxyl groups and methyl groups in the molecular structure of ACN [10,11,12]. The more hydroxyl groups, the bluer the colors, while a number of methoxyl groups are associated with red [13]. Therefore, ACN exhibits diverse hues and unique advantages such as natural pigments, good antioxidant properties, high safety, and environmental friendliness. However, the color of ACN is unstable and fleeting without any protection due to its structure transformation, which is a critical defect to realize the wide applications of ACN. As a result, it is indispensable to develop a feasible strategy to enhance the color stability of ACN.

Inspired by the preparation of Maya blue pigments, synthetic soap stone and mesoporous silica as inorganic substrates were employed to improve the stability of ACN and achieve reversible color changes [14,15]. Compared with synthetic substrates, natural clay minerals exhibit attractive advantages to design hybrid pigments with good stability due to the low-cost, abundance in nature, eco-friendliness, etc. Due to its unique layered structure, montmorillonite (Mt) was used to improve the stability of natural ACN [1,3,6,16]. Silva et al. Also prepared highly fluorescent hybrid pigments with improved color and thermal stability by encapsulating a series of models of ACN into the tunnels and grooves of sepiolite (Sep) [17]. In addition, our groups recently synthesized the reversible allochroic hybrid pigments with excellent stability by incorporation of ACN into palygorskite (Pal) [18]. However, it is well-known that different clay minerals have different morphologies, structures, chemical compositions, and properties depending on the arrangement of tetrahedral and octahedral sheets [19,20,21,22,23]. These factors might be closely related to the formation mechanism and properties of hybrid pigments composed of clay minerals and dyes. For example, Pal protected indigo from the damage of external environments by enclosing the natural dyes in its nanochannels, and thus hybrid pigments exhibited excellent sunlight resistance and chemical stability [24,25]. In the case of Sep, the involved interactions between clay minerals and dyes mainly included encapsulation of nanochannels, H-bond formation, complexing action, and the presence of dehydroindigo [26,27,28]. Furthermore, Bernardino et al. proposed that indigo molecules were constrained by the micro-channels of Pal to avoid molecular geometry rearrangement and fast energy relaxation, thus preventing from the inactivation of Maya blue [29]. It might explain why methylene blue (MB) stimulants obtained from laponite, Mt and Sep failed to exhibit the same chemical stability with MB. In a word, the dye molecules might exhibit the selectivity toward clay mineral substrates. Therefore, it is crucial to explore the effect of the possible interaction mechanism between ACN and clay minerals on the color performances of hybrid pigments.

In this study, different clay minerals were employed to prepare ACN/clay mineral hybrid pigments by combining adsorption, grinding, and heating treatment, in which ACN was extracted from blueberries. The interaction mechanism between clay minerals and ACN was comparatively investigated, while the structures and properties of the final hybrid pigments were systematically studied, especially the color response toward acidic and alkaline atmospheres. The main purpose of this work is to discuss the possible loading modes between clay mineral and natural ACN, and thus to guide the design of environmentally friendly intelligent pigments with excellent properties using natural clay minerals and ACN. In addition, the allochroic behavior of the obtained hybrid pigments to different pH values was also explored after being coated on the filter paper.

## 2. Experimental

### 2.1. Materials

Kaolinite (Kal) was taken from Longyan Kaoline Development Co., Ltd., Longyan, China. Mt was obtained from Jiashan Baishiwei Biotech Co., Ltd., Jiaxing, China. Halloysite (Hal) was provided by Zhengzhou Jinyangguang Ceramics Co., Ltd., Zhengzhou, China. Sep was supplied by Yixian Dazhi Insulation Materials Sepiolite Co., Ltd., Baoding, China. The chemical compositions of the above clay minerals are shown in Table S1. The clay minerals were ground and treated with 4% HCl (wt.%), and then filtered by passing through a 200-mesh sieve and dried. ACN was extracted from fresh blueberry fruits purchased from the local market [30].

### 2.2. Preparation of ACN/Clay Mineral Hybrid Pigments

In the experiment, 1.31 g of purified clay minerals were dispersed into 13 mL of distilled water and magnetically stirred for 30 min, and then it was slowly added to the blueberry extract under continuous stirring at 120 rpm, followed by oscillation with 190 rpm at 27 °C for 24 h in a constant temperature shaker (THZ-98A, INESA, Shanghai, China). After adsorption, the above mixture was separated by centrifugation (TDL-5C, INESA, Shanghai, China) at 4000 rpm for 10 min, and the remaining precipitate was ground in a mortar for 30 min. Finally, the obtained samples were treated at 120 °C for 4 h for subsequent testing. The prepared hybrid pigments were labeled as ACN/Kal, ACN/Mt, ACN/Hal, and ACN/Sep according to the involved clay minerals, respectively.

### 2.3. Evaluation of Environmental Stability

Thermal stability of hybrid pigments was investigated using an STA449F3 simultaneous thermal analyzer (NETZSCH-Gerätebau GmbH, Selb, Germany) in the temperature range from 35 to 800 °C at the rate of 10 °C/min under nitrogen atmosphere. The differences in chemical stability of different hybrid pigments in acidic, ethanol and basic solutions also were studied. Typically, the obtained samples (0.02 g) were dispersed into 10 mL of 1 M HCl, ethanol and 1 M NaOH, respectively, and then vibrated at 70 rpm and room temperature for 24 or 72 h in a constant temperature shaker. After centrifugation at 4000 rpm for 10 min, the supernatant was measured using a TU-1900 UV-vis spectrometer (PERSEE, Beijing, China) to calculate the concentration of the desorbed ACN from hybrid pigments. The color response of hybrid pigments in acid/base atmosphere was recorded using a Nikon D7100 camera (Nikon Corporation, Tokyo, Japan) and the colorimetric values of samples were measured by a Color-Eye automatic differential colorimeter (X-Rite, Ci 7800, Pantone Inc., Carlstadt, NJ, USA).

### 2.4. Reversible Acid/Base Allochroic Behavior

The hybrid pigments of ACN/Kal, ACN/Mt, ACN/Hal, andACN/Sep were alternatively placed in two independent atmospheres of HCl and NH_3_·H_2_O in two closed desiccators. Firstly, the obtained four samples were exposed to HCl or alkaline atmosphere, and then the hybrid pigments were transferred into the opposite atmosphere every 6 min to realize the periodical transformation of different atmospheres.

### 2.5. Color Response Analysis of pH Test Papers

Firstly, 0.1 g of ACN/Sep hybrid pigment was dispersed into 20 mL of ethanol by continuous ultrasound (Scientz-IID, Ningbo Xinzhi Biotechnology Co., Ltd., Ningbo, China) at room temperature for 10 min. Then, 1 mL of the mixed solution was uniformly dripped onto filter papers with a diameter of 2.00 cm. The pH test paper was obtained after the filter paper coated with hybrid pigment was dried. The aqueous solutions with different pH values from 1.00 to 14.00 were dripped onto the prepared dry test papers, respectively, and then the allochroic phenomenon was recorded.

### 2.6. Characterizations

The X-ray diffraction patterns (XRD) were obtained from X’pert PRO diffractometer (PANalytical Co., Almelo, The Netherlands) along with Cu-Ka radiation at 40 kV and 30 mA, the diffraction data of samples were obtained from 3° to 80° at a scanning speed of 2° per minute. The Fourier transform infrared (FTIR) spectra of series samples were recorded in the range of 4000–400 cm^−1^ on a Nicolet NEXUS FTIR spectrometer (Thermo Electron Corp., Somerset, NJ, USA) using KBr pellet. The zeta potentials of hybrid pigments were evaluated by Malvern Zetasizer Nanosystem (Malvern Instruments Ltd., Melvin, UK) with a 633 nm He-Ne laserir radiated. The morphologies of samples were observed by a field emission scanning electron microscopy (FESEM, JSM-6701F, JEOL, Tokyo, Japan). The surface area and pore volume of samples were measured at −196 °C with N_2_ as an adsorbate using the Accelerated Surface Area and Porosimetry System (ASAP2020-M, Micromeritics Instrument Corp, Atlanta, GA, USA). The colorimetric values of all hybrid pigments were calculated on a Color-Eye automatic differential colorimeter (X-Rite, Ci 7800, Pantone Inc., Carlstadt, NJ, USA) according to the Commission International del’Eclairage (CIE) 1976 L^*^, a^*^, b^*^ colorimetric method, where L^*^ (0-black/100-white), a^*^ (negative-green/positive-red) and b^*^ (negative-blue/positive-yellow), C^*^ (chroma) represented the saturation of color and *h*° was the hue angle. All measurements were taken three times for each type of hybrid pigment. Thermal gravimetric analysis (TGA) was obtained on an STA449F3 simultaneous thermal analyzer (NETZSCH-Gerätebau GmbH, Selb, Germany). The UV-Vis spectra of the eluate were obtained on TU-1900 UV-vis Spectrometer (PERSEE, Beijing, China). The UV-vis diffuse reflectance spectra were also collected from X-Rite, Ci7800, and the corresponding absorption spectra were also transformed by the obtained by the Kubelka–Munk method. The chemical compositions of clay minerals were collected from E3X-ray fluorescence spectrometer (PANalytical, Almelo, Netherlands).

## 3. Results and Discussion

### 3.1. Characterization of ACN/clay Mineral Hybrid Pigments

**Figure 1:**
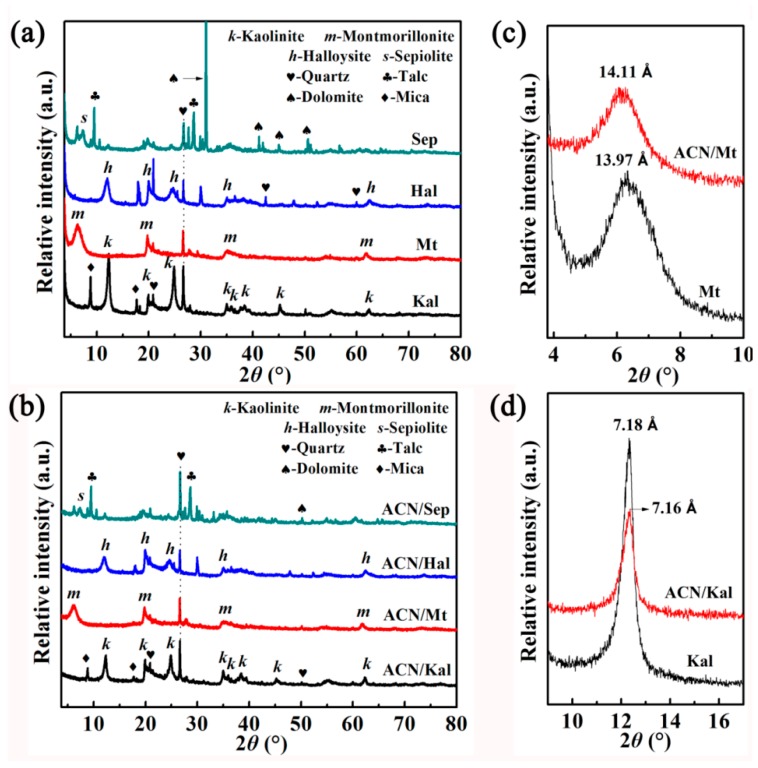
XRD patterns of (**a**) the raw clay minerals, (**b**) ACN/clay mineral hybrid pigments, the corresponding partial enlarged view of (**c**) Mt and (**d**) Kal before and after incorporation of ACN.

The XRD patterns of the raw clay minerals (Kal, Mt, Hal, and Sep) and hybrid pigments are depicted in Figure 1a,b, respectively. Two reflections at *2θ* = 8.82° and 17.68° were attributed to mica, while the characteristic reflections of talc appeared at 9.54° and 28.68°. The diffraction peaks of dolomite were found at *2θ* = 31.04°, 41.22°, 45.01°, and 50.62°. Furthermore, the characteristic peaks of quartz were observed at *2θ* = 20.84°, 26.63°, 42.48°, 50.13°, and 59.98° [23,31,32,33]. The typical diffraction peaks of Kal (*2θ* = 12.32° and 24.88°), Mt (*2θ* = 6.32°), Hal (*2θ* = 12.04° and 24.72°) and Sep (*2θ* = 7.39°) were all observed in their XRD patterns [31,34,35,36,37]. After incorporation of ACN, the characteristic peaks of clay minerals still could be observed indicating that their crystal structures remained during the preparation of hybrid pigments, and the basal distances of the characteristic diffraction peaks of Kal, Hal, and Sep did not change significantly. In the case of Mt, the basal distances of (100) plane increased from 13.97 Å to 14.11 Å (Table S2, Figure 1c,d). It suggested that a small number of ACN were intercalated into the interlayer of Mt, while they only were adsorbed on the surface of Kal [1,6,38]. Furthermore, the grinding and heat treatment process might slightly break the stacking order between tetrahedral and octahedral sheets to some extent, resulting in the intensity peaks of clay minerals weakened after incorporation of ACN molecules [39,40].

**Figure 2:**
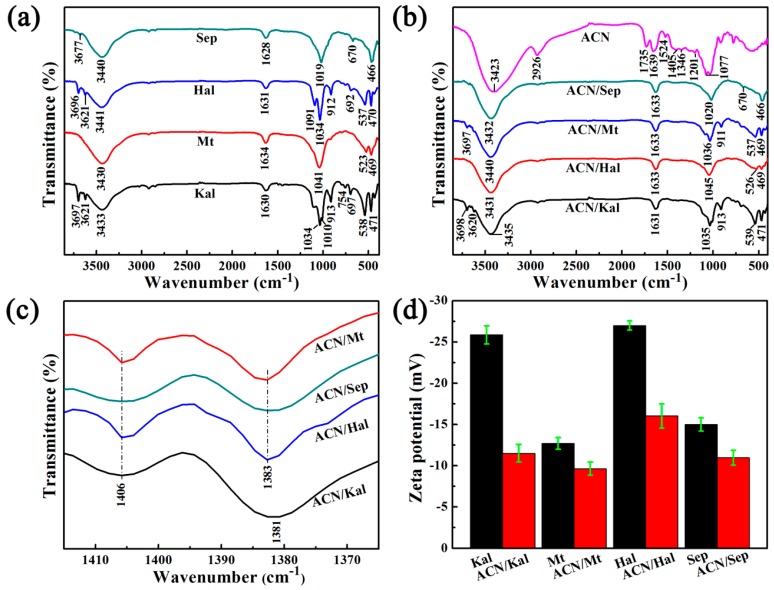
FTIR spectra of (**a**) the raw clay minerals, (**b**) ACN/clay mineral hybrid pigments and extracted ACN, (**c**) the partial enlarged view of above hybrid pigments, and (**d**) Zeta potentials of the raw clay minerals and hybrid pigments.

FTIRs pectra of Kal, Mt, Hal, Sep, and the corresponding hybrid pigments are shown in Figure 2. The absorption bands at around 3700–3500 cm^−1^ and at ~3400 cm^−1^ were mainly ascribed to the stretching vibration of O-H groups belonging to Al(Fe,Mg)-OH and H_2_O (zeolitic H_2_O) [26,36,41,42,43], bound OH_2_ [44], structural OH, and adsorbed water [31,45], respectively (Figure 2a). The bending vibration of H_2_O was located at around 1630 cm^−1^. The peaks at about 1100–1000 cm^−1^ and 475–800 cm^−1^ were originated from the Si-O bending and stretching vibrations of the tetrahedral sheets, while the bands of octahedral Al-Al-OH bending vibrations were found at around 910 cm^−1^ [46,47]. The absorption bands at 3500–2900 cm^−1^ and at 1735 cm^−1^ of the extracted ACN corresponded to the O-H groups stretching vibration and the C=O groups bending vibration, respectively (Figure 2b). Three bands appeared at 1649 cm^−1^, 1524 cm^−1^, and 1405 cm^−1^ were all attributed to the C=C stretching vibration of aromatic rings. The observed bands at 1346 cm^−1^ and 1077cm^−1^ were associated with the deformation of phenols C-O angular deformations and aromatic ring C-H deformation. In addition, a peak presented at 1201 cm^−1^ was assigned to stretching vibration of pyran rings [1,5,8]. It was worth noting that the new bands from FTIR spectra of hybrid pigments were observed at about 1406 cm^−1^ and 1383 cm^−1^ compared with that of clay minerals, which were attributed to the characteristic bands of ACN molecules (Figure 2c), revealing that natural ACN dyes were successfully loaded on different clay minerals. In addition, the vibration of O-H groups of Sep shifted from 3440 cm^−1^ and 1628 cm^−1^ to 3432 cm^−1^ and 1633cm^−1^, respectively, which might be ascribed to the electrostatic interaction and hydrogen bond between ACN and Sep [5,26].

Furthermore, the involved clay minerals in this research were all negatively charged (Figure 2d and Table S3). The zeta potential of Kal and Hal drastically increased from −25.87 mV to −11.5 mV and −27.00 mV to −16.03 mV with the incorporation of ACN, respectively, and the zeta potential increased by 55.6% and 40.6%, respectively. By contrast, Mt and Sep presented the lowest increase in the zeta potential compared with that of Kal and Hal. Therefore, it suggested that the cationic ACN dyes were successfully loaded on clay minerals due to electrostatic interaction. The inner surface of Hal nanotubes was positively charged while the interlayer of Kal had a lower cation exchange capacity, suggesting that dyes might be adsorbed on the external surface of Kal and Hal [19,48]. Combined with the results of XRD, it indicated that ACN molecules might mainly enter the interlayer of Mt and the nanochannels of Sep, respectively.

**Figure 3:**
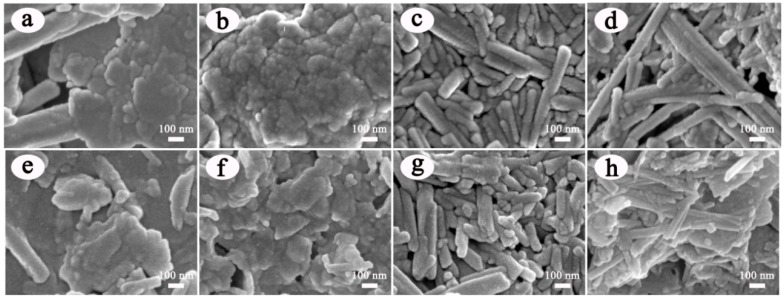
SEM images of (**a**) Kal, (**b**) Mt, (**c**) Hal, (**d**) Sep, (**e**) ACN/Kal, (**f**) ACN/Mt, (**g**) ACN/Hal and (**h**) ACN/Sep.

The surface morphologies of clay minerals are characterized by SEM before and after incorporation of ACN. As illustrated in Figure 3, the main morphological features of the raw clay minerals were clearly observed, in which Kal and Mt were the lamellar structure. In Figure 3a, the accompanied one-dimensional morphologies in Kal were belonged to the associated Hal based on XRD patterns [19], which were also similar to the shape of Hal presented in Figure 3c. After introduction of ACN, the morphologies of the obtained ACN/Kal (Figure 3e), ACN/Mt (Figure 3f), ACN/Hal (Figure 3g), and ACN/Sep (Figure 3h) were consistent with that of the corresponding clay minerals without the obvious changes, and some slight changes in the sizes of hybrid pigments might be due to the grinding procedure.

The loading of ACN on clay minerals might lead to changes in their pore structure parameters. As listed in Table 1, the lamellar Mt had the largest *S_BET_* and *S_ext_* values of 100.10 m^2^/g and 85.20 m^2^/g compared with other clay minerals, respectively. The *V_total_* value of Hal was highest (0.1731 cm^3^/g) among the involved clay minerals. It was obvious that the pore structure parameters of hybrid pigments greatly decreased after incorporation of ACN. In order to verify the reason for the decrease in the pore structure parameters, the clay minerals without addition of ACN were treated by the similar procedures for preparation of hybrid pigments. It was found that the grinding process affected the area and volume of clay minerals without ACN, but the successful loading of ACN further intensified this phenomenon (Table S4). In addition, the decrease of *S_ext_* also indicated that some ACN molecules were adsorbed on the surface of clay minerals. Therefore, it confirmed that the decrease in the pore structure parameters was ascribed to the synergistic effect of the loading of dye molecules and the mechanical force during grinding.

### 3.2. Properties of ACN/Clay Mineral Hybrid Pigments

#### 3.2.1. Color Properties

**Figure 4:**
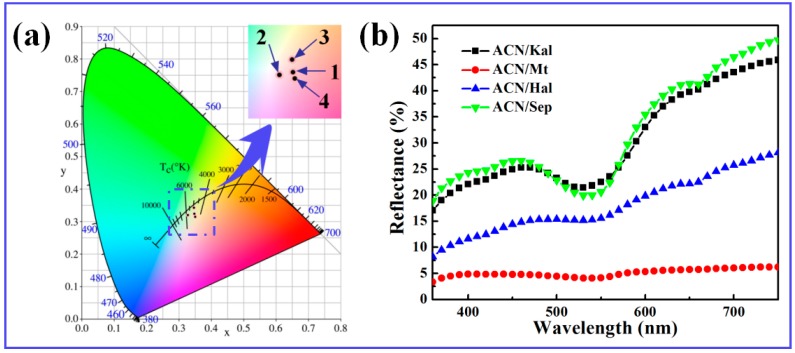
(**a**) Chromatic CIE coordinates and (**b**) UV-vis diffuse reflectance spectra of hybrid pigments (1: ACN/Kal; 2: ACN/Mt; 3: ACN/Hal; 4: ACN/Sep).

As shown in Table 2, the values of L^*^ of Kal and Sep were above 80, which were higher than those of Mt and Hal. It suggested that different clay minerals presented different colors, which was mainly related to their compositions, especially Fe_2_O_3_ (Table S1). Thus, the colors of clay minerals also affected the colors of the resulting hybrid pigments. Compared with the raw clay minerals, the L^*^ and b^*^ values of the corresponding hybrid pigments decreased due to the successful loading of ACN molecules. However, the values of a^*^ increased significantly due to the incorporation of ACN, which was related to the species of ACN in an acidic medium preventing ACN molecules from degradation. Among them, ACN/Sep exhibited the highest a^*^ and C^*^ values of 17.37 and 17.38, respectively, and the lowest *h^°^* value of 75.63, indicating ACN/Sep presented the most vivid red color. It also could be clearly seen that the color of ACN/Sep was much closer to the red area than other hybrid pigments (Figure 4a). This might be attributed to the encapsulation of ACN molecules in nanochannels and grooves of Sep, as previously reported [28].

Furthermore, the characteristic absorption bands in the range of 520–550 nm representing red were due to the presence of ACN molecules (Figure 4b). However, the L^*^ value of ACN/Mt was 25.72 and the whiteness was very low, as a result, the light was almost completely absorbed in the whole visible region. This phenomenon might be related to the compositions of Mt, which might lead to the oxidation of ACN during grinding. In order to confirm this assumption, the control experiments were conducted by incorporating Fe^3+^ and Fe^2+^ into ACN, respectively. As shown in Figure S1, it was clearly found that Fe^3+^ had an obvious effect on the stability and the color of ACN, which was also consistent with the references [49,50,51]. Therefore, the color phenomenon of ACN/Mt was mainly ascribed to the Fe species in Mt. In addition, the strength of the absorption bands also related to the degree of redness and the content of the loaded organic molecules, and the prepared hybrid pigment derived from Sep presented the maximum loading of ACN and the best color properties.

#### 3.2.2. Environmental Stability of ACN/Clay Mineral Hybrid Pigments

**Figure 5:**
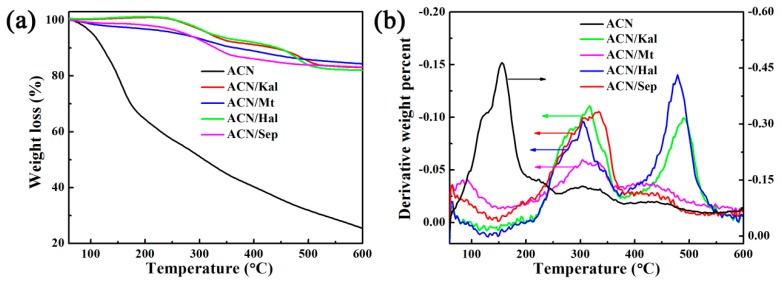
TGA (**a**) and DTG (**b**) curves of ACN extracted from blueberry and hybrid pigments.

Since natural ACN molecules are susceptible to temperature, TG and DTG curves of hybrid pigments and the extracted ACN were performed to study their thermal stability (Figure 5). The maximum degradation temperatures of ACN for ACN/Kal, ACN/Mt, ACN/Hal, and ACN/Sep were 316, 303, 303, and 338 °C, respectively, which were significantly improved compared with that of the extracted ACN of 161 °C (Figure 5a,b). As depicted in Figure S2, Kal, Mt, Hal, and Sep had no obvious mass loss before 300 °C, which was consistent with the previous reports [19,34,52]. Thus, the mass loss of above hybrid pigments at around 300 °C was attributed to the decomposition of ACN molecules, and the mass losses of ACN/Kal, ACN/Mt, and ACN/Hal were about 8.15%, 6.80%, 7.27%, respectively. In the case of raw Sep, the mass loss of structural water that occurred between 250 and 350 °C was about 0.79% [41,53,54]. Therefore, the mass loss of ACN/Sep at near 338 °C was 10.31%, suggesting Sep toward ACN exhibited the maximum loading compared with other clay minerals, which was also consistent with the result of CIE analysis. Regardless of the incorporation of any clay minerals, the thermal stability of ACN could be improved. What’s more, the structure of Sep was similar to that of Pal, and it also could encapsulate the natural dyes with a size matched with their sizes of nanochannels by electrostatic and hydrogen bonding interactions [26,27]. Thus, Sep provided a better shielding effect than other clay minerals (e.g., Kal, Mt and Hal), resulting in the highest maximum degradation temperature accompanied by better thermal stability.

**Figure 6:**
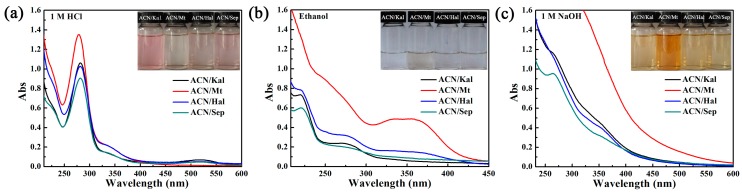
Spectra and digital images of the ACN solutions desorbed from the hybrid pigments after being treated with (**a**) 1 M HCl, (**b**) ethanol and (**c**) 1 M NaOH for 24 h, respectively.

The chemical stability of the obtained hybrid pigments is measured by exposing them to various media including 1M HCl, anhydrous ethanol and 1 M NaOH for 24 h. According to the UV-vis spectra and the colors of the supernatants in Figure 6, the following conclusions could be drawn: (1) The characteristic absorption peaks of the supernatants after hybrid pigments were treated with above three solvents were observed, which were in line with that of the extracted can dissolve in above three media (Figure S3). The colors of the three supernatants were pink, colorless and light yellow, respectively, which corresponded to three structures of ACN of the flavylium cations, carbine pseudo-bases and chalcone, respectively [12,13,55,56]. (2) The typical peaks intensity of the supernatant from ACN/Sep was the lowest when four hybrid pigments were exposed to 1 M HCl, ethanol or 1 M NaOH solution for 24 h. It revealed that ACN/Sep presented the optimum environmental stability due to the unique nanochannels for protecting the ACN molecules from external conditions. In order to further study the chemical stability of hybrid pigments, the obtained solids, after being treated with HCl, NaOH, and ethanol, were collected for color analysis. As listed in Table 3, the color parameters of samples had no significant difference before and after being immersed in acid and ethanol. However, the a^*^ values of hybrid pigments after being treated using NaOH were greatly reduced compared with the original hybrid pigments, while the b^*^ values increased due to the structural transformation of ACN loaded on clay minerals from flavylium cations to chalcone. Therefore, the lower absorbance of the supernatants and the better color parameters of hybrid pigments indicated that incorporation of clay minerals, except for Mt, obviously improved the chemical stability of natural ACN inorganic solvent, acidic and alkaline solutions.

#### 3.2.3. Reversible Acid/Base Allochroic Behavior in Different Atmospheres

**Figure 7:**
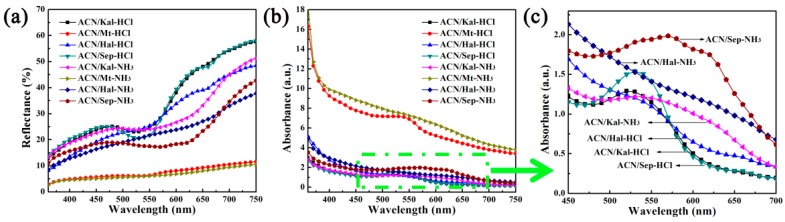
(**a**) UV-vis diffuse reflectance spectra, (**b**) UV-vis spectra, and (**c**) the partial enlarged view of UV-vis spectra of hybrid pigments after being exposed to acid and alkaline atmospheres for five cycles, respectively.

It is well-known that the color of natural ACN molecules can change in the solutions with the change of pH values. However, there are few studies on the color change of ACN/clay mineral hybrid pigments in acid/base atmospheres. In this study, a certain amount of ACN/clay mineral hybrids were alternately exposed to two desiccators containing hydrochloric acid and ammonia, and the color changes of samples were observed and analyzed. As shown in Table 4, the chromaticity parameters off our samples changed as they were converted from HCl to NH_3_ atmosphere. By contrast, it was obvious that ACN/Kal and ACN/Sep presented the most obvious color changes, and the same trend was also found from the photographs and a^*^ values, and the hybrid pigments were pale pink and steel gray in HCl and NH_3_ atmospheres, respectively. Although the color change of ACN/Mt was almost invisible due to the lowest L^*^ value and the darkest whiteness, the chromaticity measurements still showed that a^*^ value decreased slightly from 4.52 to 2.46 as the sample was transferred from acidic to alkaline atmosphere. In the case of ACN/Hal, it presented a pale red and a light yellow color after being exposed to HCl and NH_3_ atmospheres, respectively, which also could be confirmed from the diffuse reflectance spectra (Figure 7a). More importantly, the color changes of hybrid pigments in different atmospheres were reversible and could be recovered within four min every time. Interestingly, this reversible color transformation occurred at least five cycles remaining the original color (Figure S4).

**Figure 8:**
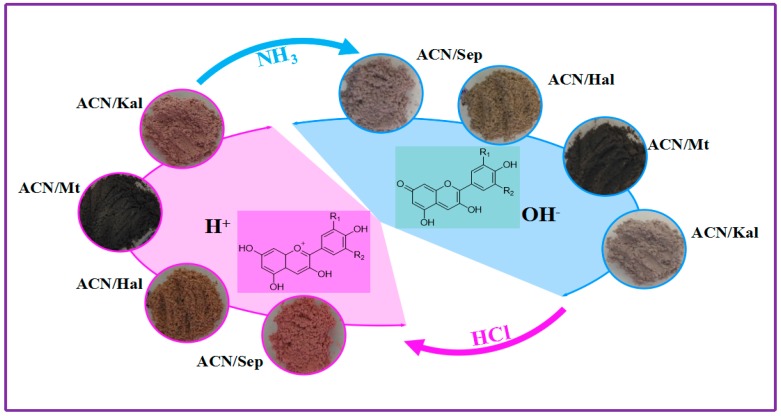
Images and scheme of the color change of ACN/Kal, ACN/Mt, ACN/Hal, and ACN/Sep in acidic and alkaline atmospheres in sequence (R1, R2 = H, OH, OCH_3_).

Figure 7b shows the absorption spectra transformed from reflectance spectra of the hybrid pigments after being exposed to HCl and NH_3_ atmospheres in turn. It was found that the absorption bands red-shifted as hybrid samples were transferred from acidic to alkaline atmosphere from the absorption spectra and partially enlarged view. The most obvious shift of absorption bands was observed from that of ACN/Sep, which was shifted from 530 to 570 nm. This phenomenon might be attributed to the structural transformation of ACN molecules loaded on clay minerals [14]. As shown in Figure 8, the color change was attributed to the structural transformation of ACN, including the number of –OH and methoxyl groups that affected conjugated double bonds of the skeleton [13]. The presence of H^+^ in HCl atmosphere resulted in the structural transformation of ACN into the flavylium cations, which was represented by the increase of a^*^ values. Furthermore, the color change in alkaline environment could be explained by that the generation of OH^−^ due to the moist NH_3_, leading to the transformation of flavylium cations to quinonoidal base.

In addition, the acid/base color changes of the prepared hybrid pigments were also studied after being sprayed on filter paper taking ACN/Sep as an example. As depicted in Figure S5, different color responses were observed after test papers were treated with the solutions with different pH from 1.00 to 14.00. It was obvious that the pH test paper became pink at pH 1.00 and pH 2.00, and then the color gradually faded and became dark purple as the pH increased from pH 3.00 to pH 11.0. In pH 12.0 and 13.0, it gradually turned pale blue, and then transform directly to yellowish-green when pH reached to 14. Interestingly, the reversible color-change behavior also could be achieved after being treated with base solution and acid solution in turn for two cycles (Figure S6).

## 4. Conclusions

In this study, the acid/base reversible allochroic hybrid pigments with different colors were successfully prepared by incorporation of ACN on different types of clay minerals. After incorporation of clay minerals, the chemical and thermal stability of natural ACN were clearly enhanced either the two-dimensional Kal and Mt or one-dimensional Hal and Sep. Due to the differences in the compositions of clay minerals and the possible interaction mechanism between ACN and clay minerals, the prepared hybrid pigments presented different color properties, especially ACN/Mt. By contrast, ACN/Sep hybrid pigment presented the optimum vivid color properties, thermal stability, and chemical corrosion resistance due to the unique nanochannels of Sep for loading of ACN. Under HCl or NH_3_ atmosphere, the same results were obtained during reversible color change of hybrid pigments derived from the structural transformation. Furthermore, a self-made pH test paper prepared by spraying ACN/Sep hybrid pigment on filter paper also obviously exhibited the color change phenomenon upon exposing in different pH solutions. Therefore, it is expected to develop a facile strategy for preparation of pH indicator combining clay minerals and natural plant pigments.

## Figures and Tables

**Table 1:** Structural parameters of the raw clay minerals and hybrid pigments.

Samples	*S_BET_* (m^2^/g)	*S_micro_* (m^2^/g)	*S_ext_* (m^2^/g)	*V_total_* (cm^3^/g)
Kal	20.71	-	23.83	0.0408
ACN/Kal	12.66	-	19.36	0.0232
Mt	100.10	14.91	85.20	0.1191
ACN/Mt	29.41	-	47.76	0.0667
Hal	65.67	-	80.73	0.1731
ACN/Hal	30.41	-	34.14	0.1261
Sep	61.29	-	72.97	0.0857
ACN/Sep	25.89	-	41.08	0.0537

**Table 2:** Color parameters of the raw clay minerals and hybrid pigments.

Color Parameters	Kal	ACN/Kal	Mt	ACN/Mt	Hal	ACN/Hal	Sep	ACN/Sep
L^*^	88.85	58.02	78.64	25.72	79.74	48.09	84.20	58.12
a^*^	0.17	13.21	0.55	4.57	4.55	5.89	0.25	17.37
b^*^	4.59	2.50	10.60	-0.56	11.51	6.11	6.30	0.47
C^*^	4.59	13.44	10.61	4.60	12.38	8.49	6.30	17.38
*h^°^*	87.85	78.77	87.75	81.08	85.31	83.40	87.84	75.63

**Table 3:** Color parameters of hybrid pigments after being treated with 1 M HCl, ethanol, 1 M NaOH for 24 h, respectively.

Samples	After Being Treated with HCl			After Being Treated with Ethanol			After Being Treated with NaOH		
	L^*^	a^*^	b^*^	L^*^	a^*^	b^*^	L^*^	a^*^	b^*^
ACN/Kal	58.18 ± 1.14	11.15 ± 0.08	4.06 ± 0.27	57.89 ± 0.04	13.74 ± 0.04	1.60 ± 0.01	74.45 ± 0.60	6.07 ± 0.20	19.76 ± 0.33
ACN/Mt	19.64 ± 0.50	4.58 ± 0.38	−0.81 ± 0.10	25.9 ± 0.54	4.16 ± 0.22	−1.10 ± 0.24	41.84 ± 1.13	6.07 ± 0.34	12.35 ± 0.79
ACN/Hal	48.96 ± 0.81	5.49 ± 0.10	7.87 ± 0.10	49.06 ± 0.66	4.16 ± 0.22	4.91 ± 0.07	69.86 ± 0.09	6.01 ± 0.04	19.53 ± 0.03
ACN/Sep	52.24 ± 0.29	16.45 ± 0.03	1.85 ± 0.04	58.02 ± 0.10	16.42 ± 0.41	−1.25 ± 0.45	67.53 ± 0.29	6.39 ± 0.06	20.68 ± 0.04

**Table 4:** Color parameters of the ACN/clay minerals before and after being exposed to HCl and NH_3_ atmospheres for five cycles.

Samples	Color Parameters in HCl			Color Parameters in NH_3_		
	L^*^	a^*^	b^*^	L^*^	a^*^	b^*^
ACN/Kal	60.54 ± 0.25	15.08 ± 0.49	8.50 ± 0.19	57.09 ± 0.16	3.34 ± 0.10	4.35 ± 0.18
ACN/Mt	31.54 ± 0.65	4.52 ± 0.14	4.98 ± 0.21	29.92 ± 0.16	2.46 ± 0.18	4.93 ± 0.35
ACN/Hal	59.20 ± 0.17	8.30 ± 0.20	14.17 ± 0.13	53.63 ± 0.03	2.48 ± 0.08	10.58 ± 0.12
ACN/Sep	59.62 ± 0.22	19.36 ± 0.15	6.00 ± 0.01	49.67 ± 0.46	1.70 ± 0.04	−0.22 ± 0.11

## Supplementary Materials

The following are available online at https://www.mdpi.com/1996-1944/12/20/3287/s1, Table S1: Chemical compositions of the clay minerals; Table S2: Crystal information of clay minerals and their corresponding hybrid pigments; Table S3: Zeta potentials of the raw clay minerals and corresponding hybrid pigments; Table S4: Pore structure parameters of the raw clay minerals after being grinded for 30 min without ACN; FigureS1: UV-Vis spectra and digital images of ACN solutions before and after being treated with Fe^2+^ and Fe^3+^, respectively; Figure S2: TGA curves of the raw Kal, Mt, Hal, and Sep; Figure S3: UV-Vis spectra and digital images of ACN solutions after being treated with (a) 1 M HCl, (b) ethanol and (c) 1 M NaOH, respectively; Figure S4: Digital images of the obtained ACN/Kal, ACN/Mt, ACN/Hal, and ACN/Sep after five acid/base cycles; Figure S5: Digital images of pH test papers at different pH (1.00–14.00); Figure S6: Digital images of pH test papers and reversible color change after being repeatedly treated with base and acid solutions in turn.
