# Supplementary material for: A Comparative Study on Color Stability of Anthocyanin Hybrid Pigments Derived from 1D and 2D Clay Minerals
Source: Materials (Basel). 2019 Oct 10;12(20):3287. doi: 10.3390/ma12203287 (PMC6829488; doi:10.3390/ma12203287)
Supplement: Supplementary file 1 [file materials-12-03287-s001.pdf]

materials

Supplementary Information

A Comparative Study on Color Stability of Anthocyanin Hybrid Pigments Derived from 1D and 2D Clay Minerals

Shue Li[1,2,3], Bin Mu[1,3,*], Xiaowen Wang[1,2,3], Yuru Kang[1,3] and Aiqin Wang[1,3,*]

1 Key Laboratory of Clay Mineral Applied Research of Gansu Province, Center of Eco-Materials and Green Chemistry, Lanzhou Institute of Chemical Physics, Chinese Academy of Sciences, Lanzhou 730000, China; seli17@licp.cas.cn (S.L.); wxw1201@163.com (X.W.); yurukang@licp.cas.cn(Y.K.)

2 Center of Materials Science and Optoelectronics Engineering, University of Chinese Academy of Sciences, Beijing 100049, China

3 Center of Xuyi Palygorskite Applied Technology, Lanzhou Institute of Chemical Physics, Chinese Academy of Sciences, Xuyi 211700, China

* Correspondence: mubin@licp.cas.cn (B.M.); aqwang@licp.cas.cn (A.W.); Tel.: +86-931-4868118 (A.W.); Fax: +86-931-4968019 (A.W.)

Table S1. Chemical compositions of the clay minerals.

Clay Minerals	Al_2O_3	Na_2O	MgO	CaO	SiO_2	K_2O	Fe_2O_3
Kal	29.28	0.04	0.47	0.02	47.02	2.44	0.47
Mt	14.69	0.95	3.91	1.76	62.36	0.27	5.08
Hal	29.49	0.07	0.39	0.08	42.56	0.85	1.37
Sep	4.76	4.93	25.66	7.87	49.35	0.93	1.39

Table S2. Crystal information of clay minerals and their corresponding hybrid pigments.

Samples	Basal Spacing of the Raw Clays			Basal Spacing of Hybrid Pigments		
	2θ (°)	d (Å)	(h k l)	2θ (°)	d (Å)	(h k l)
Kal	12.32	7.18	001	12.36	7.16	001
	24.88	3.58	002	24.87	3.58	002
Mt	6.32	13.97	001	6.26	14.11	001
Hal	12.04	7.34	001	11.98	7.38	001
	24.72	3.60	002	24.60	3.62	002
Sep	7.39	11.95	011	7.38	11.97	011

Table S3. Zeta potentials of the raw clay minerals and corresponding hybrid pigments.

Samples	Kal	Mt	Hal	Sep
Zeta potentials (mV)	−25.87	−12.70	−27.00	−15.00
Samples	ACN/Kal	ACN/Mt	ACN/Hal	ACN/Sep
Zeta potentials (mV)	−11.50	−9.64	−16.03	−10.97

Table S4. Pore structure parameters of the raw clay minerals after being grinded for 30 min without ACN.

Samples	S_{BET} (m^2/g)	S_{micro}(m^2/g)	S_{ext} (m^2/g)	V_{total}(cm^3/g)
kal	24.34	-	36.89	0.0679
Mt	81.85	-	94.87	0.1497
Hal	53.36	-	68.66	0.1899
Sep	48.39	-	51.45	0.0959

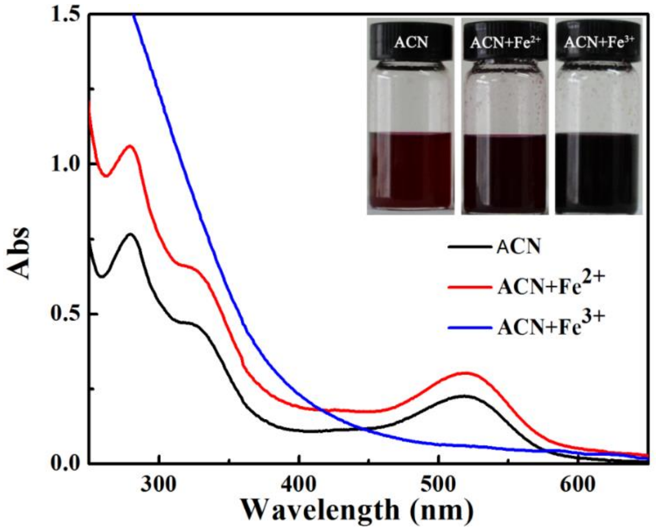

Figure S1. UV-Vis spectra and digital images of ACN solutions before and after being treated with Fe^{2+} and Fe^{3+}, respectively.

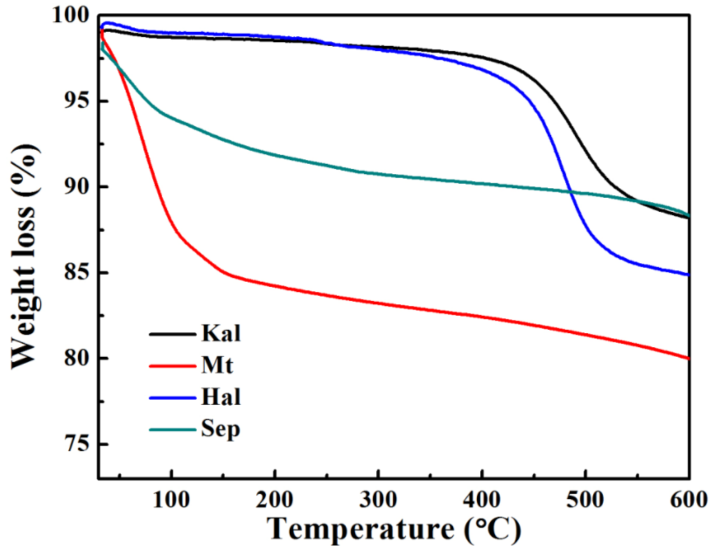

Figure S2. TGA curves of the raw Kal, Mt, Hal, and Sep.

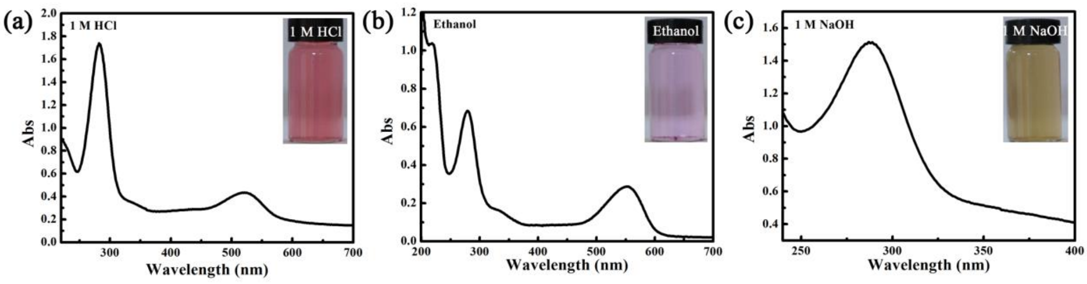

Figure S3. UV-Vis spectra and digital images of ACN solutions after being treated with (a) 1M HCl, (b) ethanol and (c) 1M NaOH, respectively.

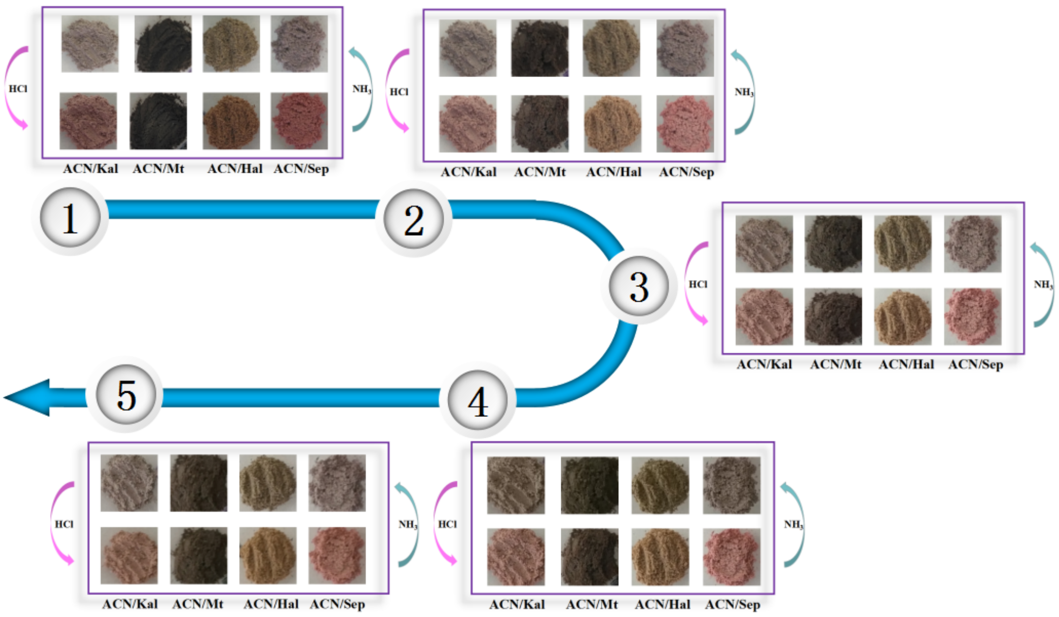

Figure S4. Digital images of the obtained ACN/Kal, ACN/t, ACN/Hal, and ACN/Sep after five acid/base cycles.

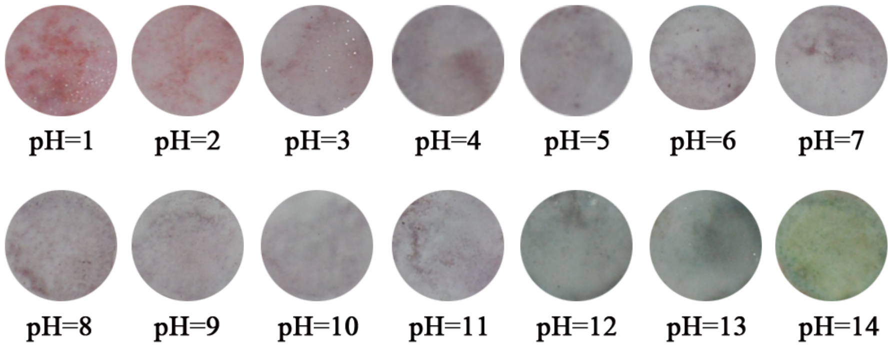

Figure S5. Digital images of pH test papers at different pH (1.00-14.00).

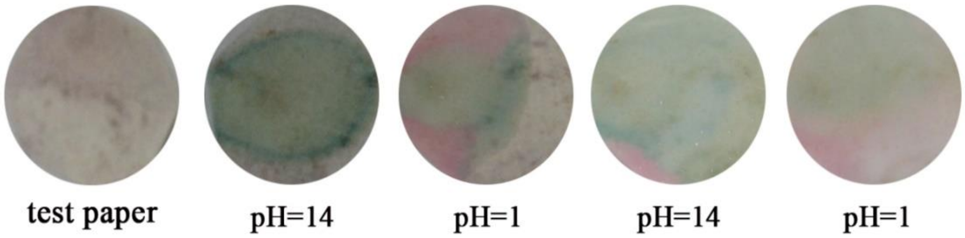

Figure S6. Digital images of pH test papers and reversible color change after being repeatedly treated with base and acid solutions in turn.
